# Genomic and transcriptomic landscape of conjunctival melanoma

**DOI:** 10.1371/journal.pgen.1009201

**Published:** 2020-12-31

**Authors:** Katarina Cisarova, Marc Folcher, Ikram El Zaoui, Rosanna Pescini-Gobert, Virginie G. Peter, Beryl Royer-Bertrand, Leonidas Zografos, Ann Schalenbourg, Michael Nicolas, Donata Rimoldi, Serge Leyvraz, Nicolò Riggi, Alexandre P. Moulin, Carlo Rivolta

**Affiliations:** 1 Department of Computational Biology, University of Lausanne, Lausanne, Switzerland; 2 Department of Genetics and Genome Biology, University of Leicester, Leicester, United Kingdom; 3 Institute of Molecular and Clinical Ophthalmology Basel (IOB), Basel, Switzerland; 4 Department of Ophthalmology, University of Basel, Basel, Switzerland; 5 Jules-Gonin Eye Hospital, Department of Ophthalmology, Fondation Asile des Aveugles, University of Lausanne, Lausanne, Switzerland; 6 Ludwig Cancer Research, Department of Oncology, University of Lausanne, Lausanne, Switzerland; 7 Charité Comprehensive Cancer Center, Berlin, Germany; 8 Experimental Pathology, Institute of Pathology, Lausanne University Hospital, Lausanne, Switzerland; Brigham and Women's Hospital, UNITED STATES

## Abstract

Conjunctival melanoma (CJM) is a rare but potentially lethal and highly-recurrent cancer of the eye. Similar to cutaneous melanoma (CM), it originates from melanocytes. Unlike CM, however, CJM is relatively poorly characterized from a genomic point of view. To fill this knowledge gap and gain insight into the genomic nature of CJM, we performed whole-exome (WES) or whole-genome sequencing (WGS) of tumor-normal tissue pairs in 14 affected individuals, as well as RNA sequencing in a subset of 11 tumor tissues. Our results show that, similarly to CM, CJM is also characterized by a very high mutation load, composed of approximately 500 somatic mutations in exonic regions. This, as well as the presence of a UV light-induced mutational signature, are clear signs of the role of sunlight in CJM tumorigenesis. In addition, the genomic classification of CM proposed by TCGA seems to be well-applicable to CJM, with the presence of four typical subclasses defined on the basis of the most frequently mutated genes: *BRAF*, *NF1*, *RAS*, and triple wild-type. In line with these results, transcriptomic analyses revealed similarities with CM as well, namely the presence of a transcriptomic subtype enriched for immune genes and a subtype enriched for genes associated with keratins and epithelial functions. Finally, in seven tumors we detected somatic mutations in *ACSS3*, a possible new candidate oncogene. Transfected conjunctival melanoma cells overexpressing mutant *ACSS3* showed higher proliferative activity, supporting the direct involvement of this gene in the tumorigenesis of CJM. Altogether, our results provide the first unbiased and complete genomic and transcriptomic classification of CJM.

## Author summary

Conjunctival melanoma is an extremely rare form of cancer of the eye that arises from melanocytes–the cells producing the protective pigment melanin–in the outmost layer of the eye: the conjunctiva. This tissue, similarly to the skin, can also be exposed to UV light radiation from the sun. We investigated the genetic background of this rare form of cancer in samples from fourteen patients, by global DNA and RNA sequencing. Our results showed that conjunctival melanoma is genetically very similar to cutaneous melanoma. More precisely, in tumor DNA we detected signs of damage caused by UV light, as well as mutations in the genes *BRAF*, *NF1* and *NRAS/HRAS*, previously described to be involved in cutaneous melanoma. Analysis of tumor gene expression also revealed similarities between these two types of cancer, some of which could be used as prognostic factors or as indicators of a patients’ response to therapy. In addition, we identified frequent somatic mutations in *ACSS3*, a gene not yet associated with either conjunctival or cutaneous melanoma, which represents a potential key player in oncogenesis of conjunctival melanoma.

## Introduction

Conjunctival melanoma (CJM) is thought to arise from melanocytes localized in an external mucosal membrane that partially covers the eye and the eyelids, the conjunctiva. CJM presents as a persistent, usually pigmented lesion on the surface of the eyeball (bulbar conjunctiva) or on the palpebral (tarsal) conjunctiva. In the majority of cases, the tumor arises from preexisting flat pigmented melanocytic proliferations, termed primary acquired melanosis (PAM) or Conjunctival Melanocytic Intraepithelial Neoplasia (C-MIN). Less frequently, it can arise from a pre-existing nevus, or even completely *de novo* (without pre-existing lesions) [1,2]. CJM is extremely rare, with an incidence rate ranging from 0.24 to 0.80 cases per million per year [3–7]. It is, however, a very recurrent tumor (recurrence is higher than 50%) [8–10] that spreads through the lymphatic system, with metastatic death occurring in 25–35% of patients within 10 years from the initial diagnosis [7,8,11–15].

In contrast to other mucosal membranes, the bulbar conjunctiva is directly exposed to the ultraviolet (UV) light radiation from the sun, suggesting a role for this agent in the tumorigenesis of CJM [16]. UV light exposure is also a known mutagenic factor in cutaneous melanoma (CM), and indeed CJM and CM share certain known risk factors, such as a lighter color of the skin and an association between incidence of disease and decreasing latitude [15,17–19]. Furthermore, the occurrence of CJM has been increasing in recent years, in a pattern that is comparable to that of CM, once again in agreement with a possible link to a sunlight-related etiology [3–5].

Unlike CM, the genetics of CJM has not been extensively evaluated. Previous genetic work on CJM only scored mutations within a panel of known genes that are frequently mutated in melanomas, or copy number variations (CNVs) detected by FISH, MLPA, array-CGH or SNP-array analysis. These studies have reported frequent mutations in known CM genes such as *BRAF*, RAS sequences (*NRAS*, *KRAS*, *HRAS*), *NF1*, *TERT*, and *KIT*, as well as patterns of CNVs resembling that of cutaneous and mucosal melanomas [20–24]. Expression analyses in CM have shown that gene expression signatures may be prognostic and that specific transcriptional patterns may be associated with tumor resistance [25], suggesting the theoretical possibility that the same could be done for CJM.

In this study, we unbiasedly assessed the landscape of genomic alterations in conjunctival melanoma by characterizing somatic single nucleotide variants (SNVs) and somatic copy number variations (CNVs) through Whole Exome Sequencing (WES) or Whole Genome Sequencing (WGS). A transcriptome profiling by RNA sequencing (RNA-seq) was also performed, allowing the characterization of transcriptional CJM subtypes and the identification of gene fusions. Overall, our results provide the first unbiased and complete genomic and transcriptomic classification of CJM and reveal that the molecular basis of CJM strongly resembles that of CM.

## Results and discussion

### Clinical description of patients and clinicopathological material

Fourteen CJM tumor tissue samples were collected from surgically-excised masses, along with the corresponding patient’s normal blood leukocytes, and screened either by WES (twelve samples) or WGS (two samples [16]). Written informed consent was obtained from all individuals enrolled in this study, and approval for human subject research was obtained from the Institutional Review Boards of all participating Institutions.

We considered samples from seven men (average age = 61.7 years) and seven women (average age = 69.6 years). Four tumor samples had tarsal conjunctiva localization and the remaining were from the bulbar conjunctiva. In ten subjects the tumor originated from either PAM/C-MIN (9/14) or from previous nevi (1/14), and in the remainder it evolved *de novo* (4/14). Two tumors were irradiated. Pigmentation was assessed in all tumor tissues and was scored as a 0–3 value (0 = no melanin pigment, 1 = slight melanin pigmentation visible at high power, 2 = moderate pigmentation visible at low power, 3 = high pigmentation readily visible at low power with dense melanin content) [26]. Over a follow-up period of 36 months (SD: 26.9 months), nine patients experienced tumor recurrence, three developed metastases, and two died of the disease. All clinical data are summarized in S1 Table.

### Elevated mutational load and presence of UV light damage signature

WGS or WES analysis identified on average 1,447 somatic SNVs (range 216–4,067) and 87 (range 49–139) small insertions or deletions (indel; all somatic SNVs and small insertions/deletions are available in S2 Table). In addition, each sample presented on average CNVs covering 33% of its genome length (range 12%-71%, S3 Table, further details in Methods). We observed that, similarly to CM, CJM is also characterized by a very high somatic mutation load, possibly the highest detected so far in tumors, composed of a median of 518 nonsynonymous somatic mutations in exonic regions. These findings are indicative of the role of UV light in the tumorigenesis of CJM and are in striking contrast with recently-obtained data from uveal melanoma (UM) [27,28], another melanoma of the eye, where only approximately 15 somatic variants per tumor are found on average. Mutational burden did not correlate with age, sex or origin of the tumor (PAM/C-MIN/nevus/*de novo*). However, lymph invasion showed a significant inverse correlation with respect to mutational load (present = 267, absent = 756, p-value = 0.02, by t-test).

**Fig 1:**
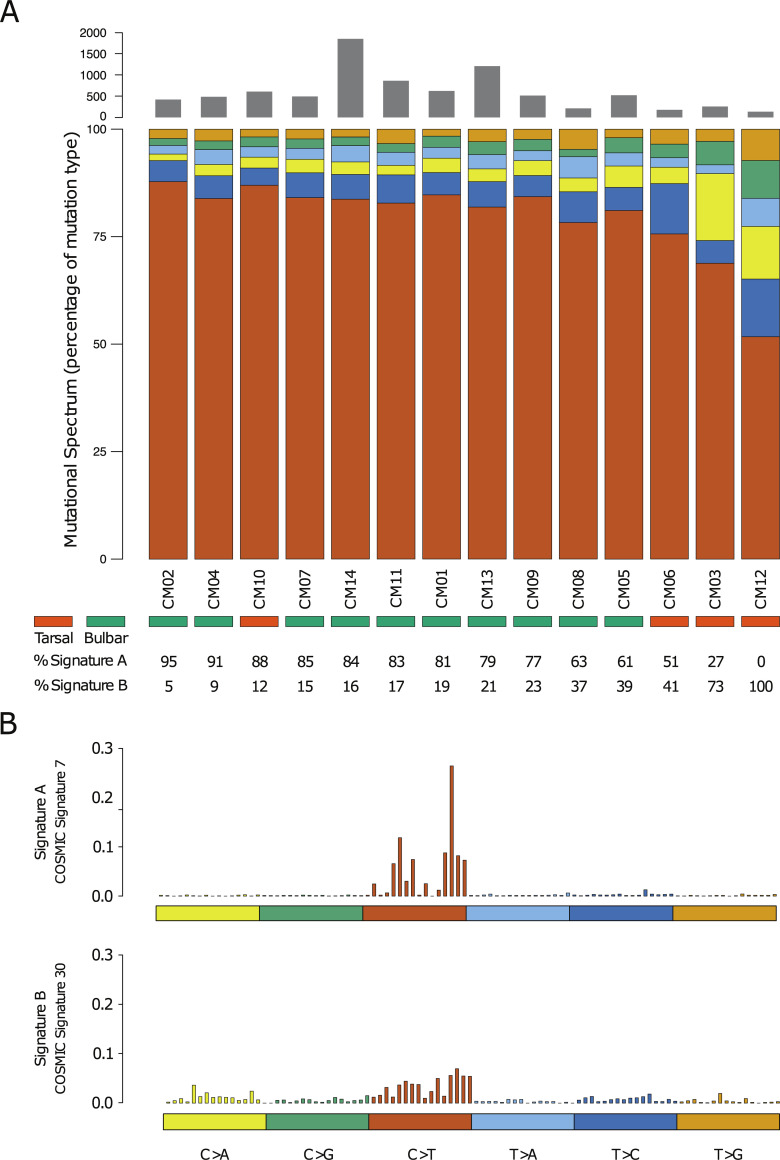
Mutational spectrum of CJM. (A) The proportions of different SNV changes show that C>T transitions dominate the mutational spectrum. Grey bars on the top indicate the number of non-neutral mutations in coding regions (see Methods). (B) Two main mutational signatures, detected in our dataset and presenting the highest correlation with COSMIC signatures 7 and 30 (v2 March 2015), are shown.

To elucidate the nature of this elevated load, we examined the mutational spectrum of this tumor by first comparing the number and contribution of different mutational events. Consistently with the presumed role of UV light in CJM tumorigenesis, in 86% of the samples C>T changes accounted for more than 70% of the total mutational load (Fig 1A). Interestingly, the three individuals with the lowest contributions of the C>T changes were samples whose tumors had a tarsal localization and therefore were less exposed to UV light. We next extracted the mutational signatures present in the data by observing the patterns of somatic mutations and by comparing them with the 30 validated signatures of mutational processes in human cancer [29]. We identified two main patterns showing the highest correlation with two COSMIC signatures: signature 7 and 30. Signature 7 is characteristic of skin cancer and resembles the mutational spectrum observed after exposure to UV light in experimental settings. Specifically, the pattern we observed resembles signature 7a (from CM, as defined by Hayward *et al*. [30]), which is most likely due to the repair of 6–4 photoproducts [31]. Signature 30 is a signature that has been observed only in a small subset of breast cancers, for which the etiology remains currently unknown (Fig 1B).

### Genetic features of tarsal CJM

The three samples with the highest contribution of signature 30 (Fig 1A) were also tumors with the lowest proportions of C>T changes, all localized tarsally. Interestingly, another patient with tarsal tumor (CM10) showed a very high contribution of signature 7 and the second highest proportion of C>T changes (Fig 1A). This patient’s tumor did not contain mutations in the APOBEC deaminases, was not enriched in APOBEC related mutations [32], and none of the APOBEC family genes showed a significant overexpression, excluding the possibility that APOBEC-mediated DNA deamination events could be the cause of the high load of C>T changes (S1 Fig). The molecular characteristics of this tumor suggest that it could have arisen from a precursor in the bulbar conjunctiva exposed to UV-light, and in fact this patient also had focal areas of pigmentation in the bulbar conjunctiva. Considering the discrepancy between the actual localization of the tumor and its genomic profile, we decided to exclude it from any further comparisons of tarsal versus bulbar tumors.

**Fig 2:**
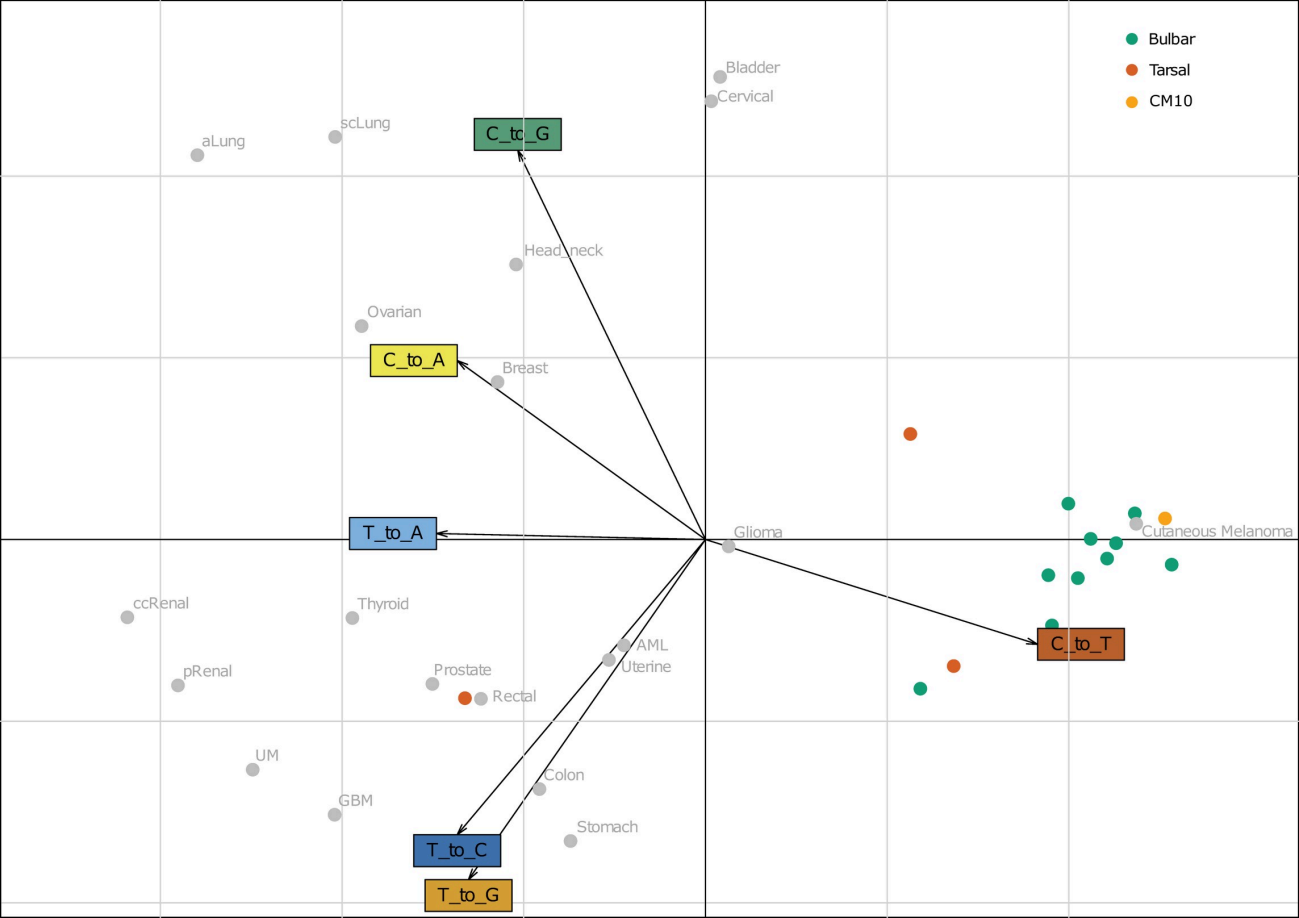
Principal-component analysis (PCA) of the mutational spectrum data. PCA is showing the similarity between CJM and CM, as well as a possible separation of tarsal vs. bulbar CJM tumors.

Next, we examined the overall load of somatic variations, specifically in relationship with tarsal vs. bulbar localization. We observed that tumors with tarsal localization had a significantly lower number of somatic variations (tarsal = 195, bulbar = 742, p-value = 0.007, by t-test) (S2 Fig). When comparing mutational spectra of these two classes of CJM by PCA (Fig 2), we also detected a substantial difference: bulbar tumors showed relatedness to CM, while tarsal tumors clustered separately. Taken together, our results suggest a different etiology for these two sub-classes of CJM.

### Genomic classification

**Fig 3:**
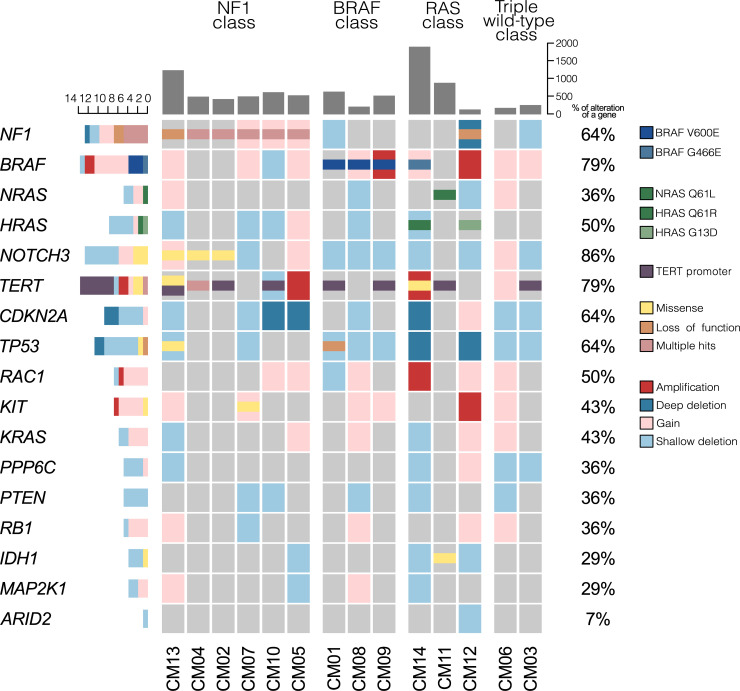
Landscape of single nucleotide and copy number variations in key driver genes in CJM, according to the 4-group genetic classification of CM. Each column represents alterations in 17 known cancer driver genes in one tumor sample; the number of non-neutral coding somatic mutations for each patient is indicated by the grey bars on the top of each column and the frequency of mutation for each gene is shown on the right side of the figure.

Assessment of mutations by MutSigCV_1.4127 revealed that no gene had higher than background mutation frequency to be considered as a potential driver. Since this is obviously not a realistic hypothesis, we concluded that the high background noise of somatic alterations that are present in CJM, as well as the limited sample size, complicated the identification of cancer driver genes by means of computer analysis alone. We therefore searched for known cancer driver events first. We screened our data for the presence of somatic alterations in driver genes from DriverDBV3 [33], and we observed that overall the detected genomic classification was indeed similar to what has been reported previously for CJM [21,34,35]. Also, it overlapped with the classification proposed by TCGA for CM subtypes, identifying four classes defined on the basis of the most frequently-mutated genes: a *BRAF*-class, a *NF1*-class, a *RAS*-class (mutation in the genes *NRAS*, *HRAS* or *KRAS*), and a triple wild-type-class [25,29]. Inactivating mutations in *NF1* (Fig 3, S3A Fig, S2 Table) were found in 7/14 samples, always present in a mutually-exclusive pattern with respect to mutations in *BRAF*. Four samples carried missense mutations in *BRAF*. In three out of these four samples we found the canonical p.V600E missense alteration, and in one sample a p.G466E mutation, also already-reported in various human cancers (S3B Fig) [25,29,36]. Mutations in genes from the RAS family were identified in three samples (1 in *NRAS* and 2 in *HRAS*), in a mutually-exclusive fashion with respect to the hotspot p.V600E in *BRAF*. These tumors carried known hotspot mutations: p.Q61R and p.G13D in *HRAS* and p.Q61L in *NRAS* (S3C and S3D Fig). In two samples, none of these major driver genes was mutated, and they were therefore classified as ‘triple wild-type’. Mutations in *hTERT* and its promoter were identified in 9/14 tumors, essentially within the same hotspots identified previously in other tumor types [37–40](S4 Table). In addition, we identified missense or loss of function mutations in less-common cutaneous melanoma drivers, such as *TP53*, *NOTCH3*, and *KIT* (Fig 3). We did not find any mutations in the known UM driver genes *GNAQ*, *GNA11*, *SF3B1*, BAP1 or *EIF1AX*, again highlighting the dissimilarity between these two ocular tumor types, whereas the landscape of mutations in CJM constitutes yet another similarity between CJM and CM. However, when combining the results of the three studies on the genetics of CJM [21,34,35] with our results and data from TCGA [25] (90 CJM and 333 CM), we found a statistically significant difference in the distribution of the four genomic classes (CJM: 31, 23, 19, 17 versus CM: 28, 166, 93, 46 for the *NF1*, *BRAF*, *RAS*, and WT classes, respectively; p-value = 4.99x10^-10^ by Pearson's Chi-squared test). While TCGA reports the *BRAF* subtype to be the most prevalent in CM, followed by the *RAS*-class and the *NF1*-class, combined results in CJM showed a different behavior. *NF1* was the gene that was found to be mutated most frequently, followed by *BRAF*, and finally the *RAS* genes [21,34].

Clinically, patients in the *NF1* class had significantly lower rates of local lymph node invasion compared to the other three classes (0:6 vs. 3:4, p-value = 0.049, by one-sided Fisher exact test). From the molecular point of view, patients in the triple wild-type class had the lowest number of exonic somatic changes (223.5 vs. 681.7, p-value = 0.008, by t-test) and the lowest contribution of the C>T changes overall (289.5 vs. 1350.5, p-value = 0.003, by t-test, S4A and S4B Fig). Again, this behavior is similar to what has been previously observed in CM [25,30]. No statistically-significant changes were observed among the molecular classes with respect to overall survival, presence or absence of metastasis, or recurrence rates.

### *ACSS3*, a new candidate oncogene

In search for additional contributors for CJM we looked for genes harboring non-synonymous mutations shared by at least three individuals. By this procedure, we identified three genes: *USH2A*, *ACSS3* and *BRAF*. The *BRAF* mutation is the canonical p.V600E missense change and therefore an already-known driver mutation, confirming the robustness of this method. The mutation in *USH2A* resulted to be a sequence artifact and therefore was discarded from our analysis. The mutation in *ACSS3* is a missense (NM_024560: c.1594C>T/p.P532S), present in a very conserved position and situated 7 amino acids away from an ATP-binding site (S5 Fig), and this alteration creates a site predicted to be phosphorylated by NetPhos [41]. We searched for the presence of p.P532S in public databases of cancer patients and cancer cell lines, and we identified this particular mutation in two cell lines from neuroblastoma and from cutaneous melanoma (Broad Institute Cancer Cell Lines Encyclopedia v18-07-2018 [42] and COSMIC v90-05-09-2019 [43]), as well as 12 additional patients with either cutaneous melanoma (8/12), skin cancer (non-melanoma) (1/12) or cutaneous squamous cell carcinoma (3/12) (cBioPortal v3.1.3 [44,45]). Furthermore, 4 additional somatic mutations in *ACSS3* were detected in our cohort of patients (S5 Fig).

**Fig 4:**
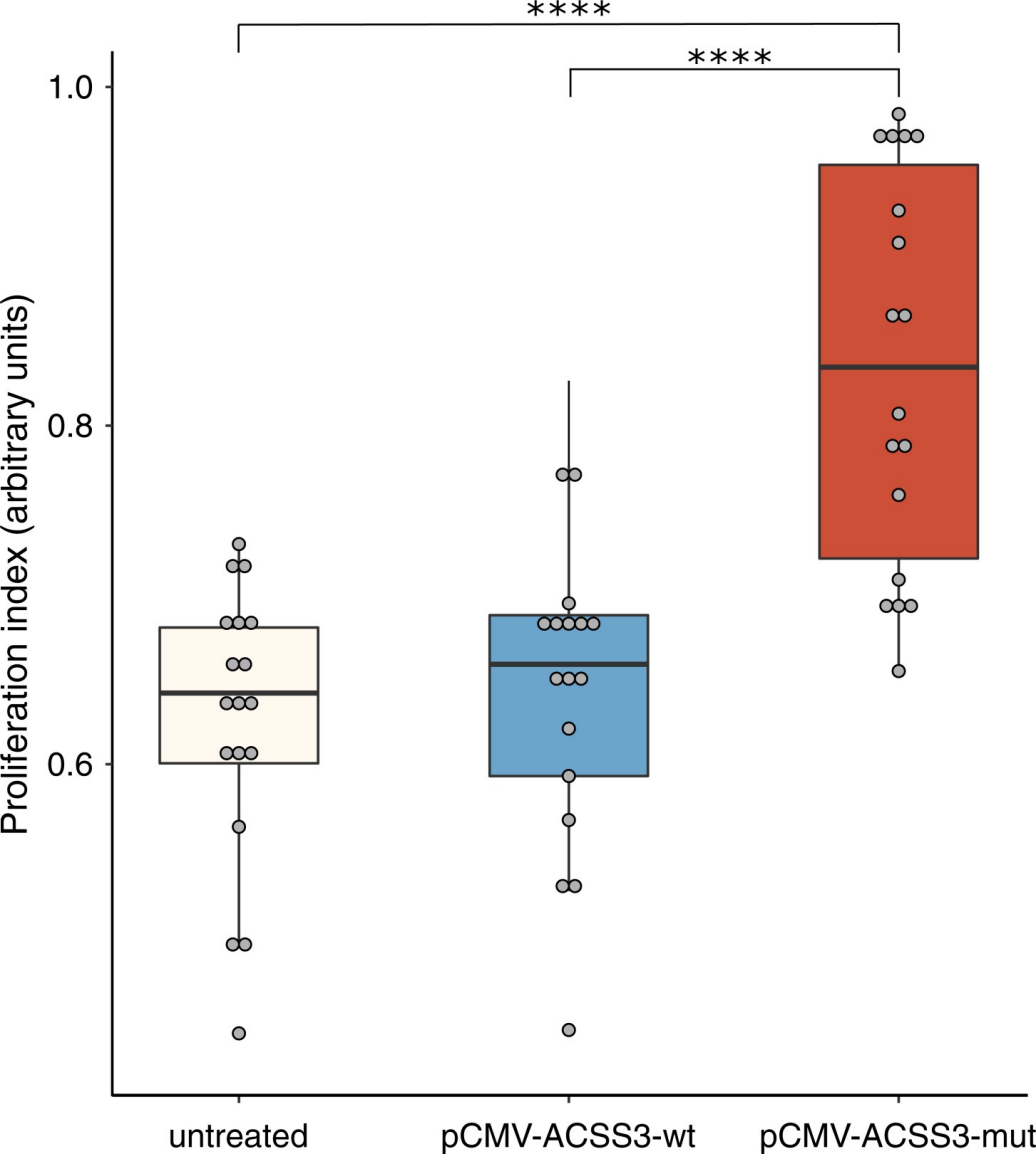
The p.P532S mutation in *ACSS3* stimulates cell proliferation. CRMM-1 cells were left untreated or transfected with a plasmid containing a wild-type *ACSS3* cDNA (pCMV-ACSS3-wt) or a cDNA containing the mutation p.P532S (pCMV-ACSS3-mut). Proliferation was assessed after 24 hours, by means of the MTT colorimetric test. Data are indicated in arbitrary units, corresponding to background-corrected absorbance values at 570 nm. Asterisks indicate significant p-values (6.91x10^-7^ between untreated and pCMV-ACSS3-mut and 3.39x10^-6^ between pCMV-ACSS3-wt and pCMV-ACSS3-mut, by t-test).

*ACSS3* is one of the three genes encoding acetyl-CoA synthetase proteins, catalyzing the synthesis of acetyl-CoA from acetate in an ATP-dependent manner. *ACSS3*, together with *ACSS1*, are mitochondrial proteins, whereas *ACSS2* is present in the cytoplasm and in the nucleus of the cell [46–48]. Multiple studies have shown that acetyl-CoA can be used by tumor cells for *de novo* lipid synthesis [49–51] and that acetyl-CoA levels are strongly associated with levels of global histone acetylation [52–54], as well as with expression of genes involved in cancer [55]. In favorable conditions of high glucose levels and presence of oxygen, acetyl-CoA is synthesized through oxidative conversion of pyruvate during glycolysis [56]. Conversely, in tumor cells glucose is preferentially converted into lactate instead of pyruvate, even in the presence of oxygen, through a process of aerobic glycolysis also known as the ‘Warburg effect’ [57,58]. The decrease in rates of oxidative phosphorylation limits the synthesis of acetyl-CoA from pyruvate, forcing the tumor cells to use alternative ways—such as through acetyl-CoA synthetase enzymes—ACSS1/2 and 3—to produce acetyl-CoA and support tumor growth [59]. Indeed, recent studies have shown that ACSS enzymes can catalyze formation of acetyl-CoA from acetate in unfavorable growth conditions in tumor cells [60,61]. In particular, *ACSS1* expression was associated with the growth and invasiveness of tumor cells in human hepatocellular carcinoma [62]. Furthermore, silencing of *ACSS2* in mouse models of liver cancer leads to dramatic reduction of tumor growth [63]. Recently, expression of *ACSS3* has also been reported in association with cancer growth and invasion in human gastric cancer and bladder urothelial carcinoma [64,65]. All these data indicate that *ACSS3* may represent an interesting candidate for tumorigenesis of CJM and that p.P532S could act as an activating driver mutation. To investigate its oncogenic impact we transfected the conjunctival recurrent malignant melanoma-1 CRMM-1 cell line (Research Resource Identifier, RRID: CVCL_M593 [66]), negative for mutations in *ACSS3*, with plasmids expressing either cDNA from the wild-type *ACSS3* gene (pCMV-ACSS3-wt) or cDNA containing the p.P532S variant (pCMV-ACSS3-mut) under the transcriptional control of the human cytomegalovirus pCMV promoter. MTT assay, assessing metabolic activity indicative of cell proliferation, was performed post-transfection and indicated a significant increase in viable and proliferating CRMM-1 cells bearing the pCMV-ACSS3-mut construct vs. its wt counterpart and vs. untransfected cells (31% and 33% increase, and p-values by t-test = 3.39x10^-6^ and 6.91x10^-7^, respectively, Fig 4). This was not simply due to increased *ACSS3* mRNA expression from the mutant plasmid vs. the wt plasmid, as we could ascertain by plasmid-specific Q-PCR (*ACSS3* expression from pCMV-ACSS3-mut vs. pCMV-ACSS3-wt = 89 ± 14 vs. 79 ± 5 arbitrary units, respectively, p-value = 0.39, by t-test), indicating indeed that the effect was consequent to the presence of the p.P532S mutation.

### Copy number variations profile

**Fig 5:**
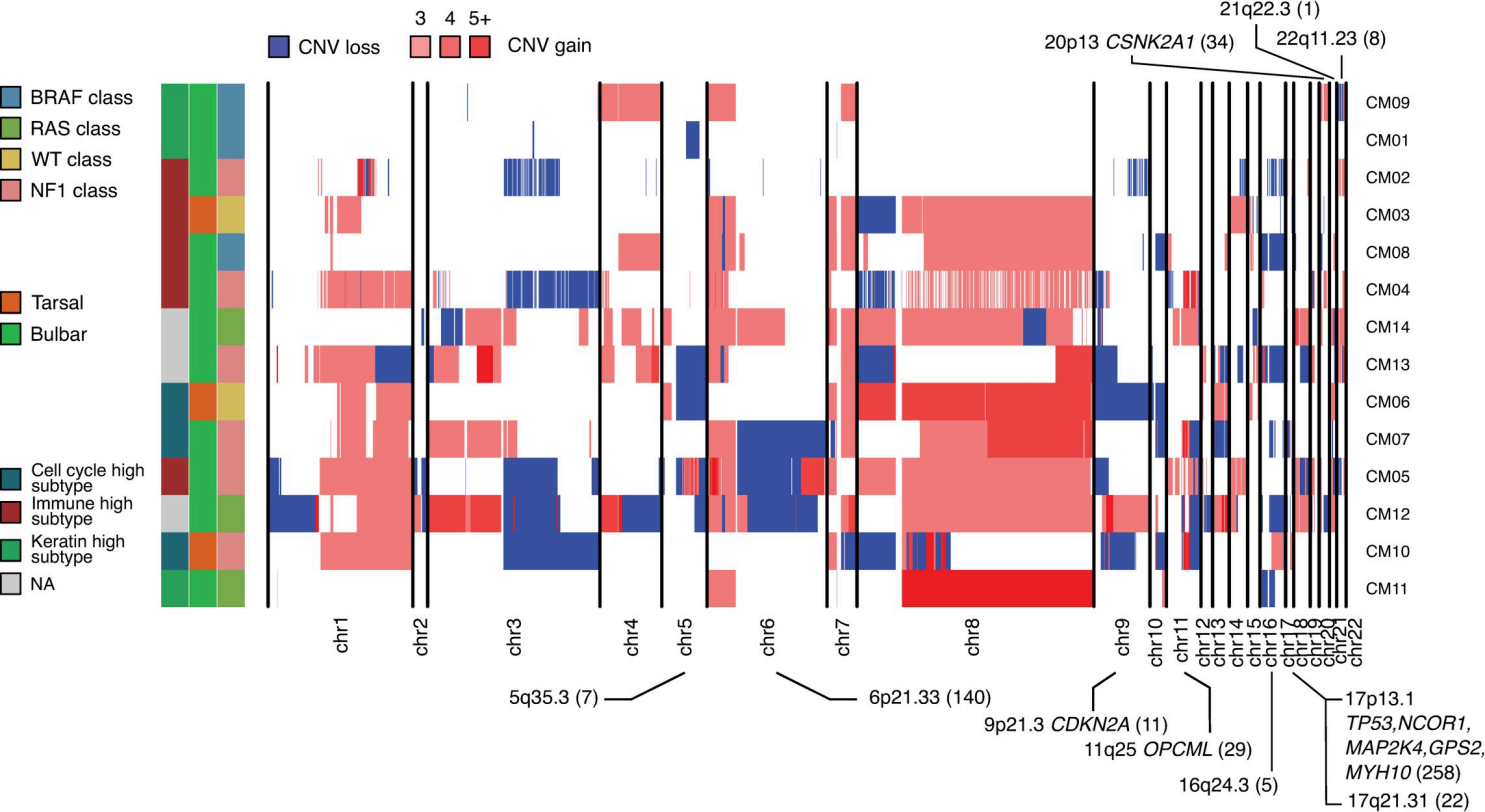
Landscape of CNVs in CJM. Sub-chromosomal level CNVs are shown, in relationship to specific characteristics of each tumor.

Somatic copy number variations (CNVs, S5 Table) were detected using CNVkit 0.9.5 [67,68] with default settings, from paired tumor and normal tissues (further details in Methods). Large CNVs impacting entire chromosomal arms are depicted in Fig 5. Significant arm amplifications were: 7p and 7q (6/14 and 8/14; amplification q-value = 5.3x10^-3^ and 4.32x10^-7^, respectively, by GISTIC 2.0), also detected in CM [69] but absent in UM [28], 8p (3/14, q-value = 3.8x10^-3^), 8q (8/14, q-value = 1.2x10^-6^) and 6p (10/14, q-value = 1.2x10^-6^). Significant arm deletions were impacting both arms of chromosome 19 (19p: 7/14, q-value = 2.83x10^-4^ and 19q: 7/14, q-value = 2.05x10^-5^) and 9p (4/14, q-value = 2.9x10^-2^). The overall pattern of CNVs overlaps with those detected previously in CJM [20,22]. In addition, we identified ten significantly-deleted focal regions (with q < 0.25) (Fig 5, S6 Fig), including the 16q24.3 region (3/14, q-value = 0.04) containing the *MC1R* gene, as well as 5q35.3 (3/14, q-value = 0.08) and 9p21.3 (3/14, q-value = 0.01), encompassing the known tumor suppressor gene *CDKN2A*, also detected in CM. The focal region 17p13.1 (deleted in 3/14, q-value = 0.14) contained well-known cancer driver genes such as *TP53*, *NCOR1M*, *MAP2K4*, *GPS2*, and *MYH10*. Interestingly, we did not detect the previously-discovered 10q11.21–26.2 deletion associated with metastasis in CJM [22]. Comparison of the proportions of CNV detected for both large and focal events in the four molecular classes defined above showed that *BRAF*-class tumors had the lowest proportion of their genomes affected by CNV [18% (*BRAF*) vs. 36.9% (not-*BRAF*), p-value = 0.01, by t-test, S3 Table].

### Transcriptomic classification

**Fig 6:**
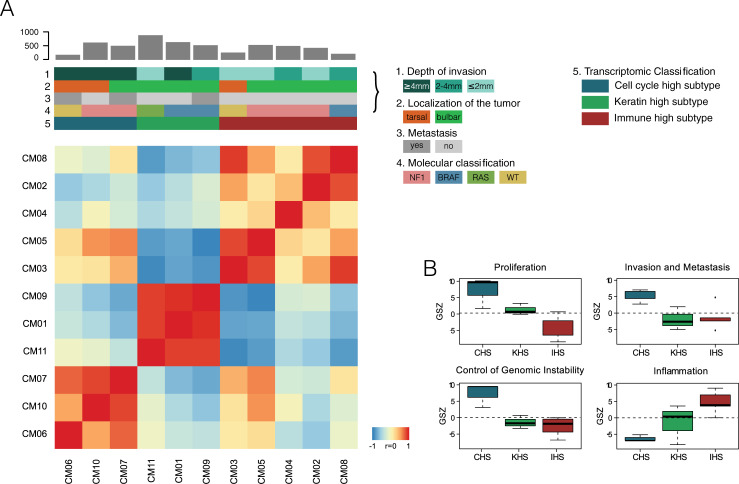
Transcriptomic classification of CJM. (A) Heatmap depicting pairwise similarity (Pearson’s correlation coefficients) between the Self Organizing Maps (SOM) portraits of single patients, highlighting the presence of three distinct clusters of samples, based on gene expression similarity (CM06-10-07, CM11-01-09, and CM03-05-04-02-08). CM gene sets mapped to the data allow for the classification of CJM into an “cell cycle high subtype”, a “keratin high subtype”, and a “immune high subtype”. Grey bars on the top of each column indicate the number of non-neutral coding somatic variants in each patient. (B) Overexpression profiles in selected hallmarks of cancer gene sets, scaled in units of the gene set Z-score in the three clusters.

Due to reduced availability of primary material, total RNA was extracted from tumor cells in 11 of the 14 CJM patients. RNA sequencing-based analysis showed three main transcriptomic clusters, according to Pearson’s correlation pairwise similarity analysis of Self Organizing Maps (SOM) portraits (Fig 6, S7 Fig). These clusters were characterized based on the GO ‘biological process’ set, as follows: (1) DNA repair, DNA replication and cell cycle, (2) immune system response and (3) keratinization, cornification and cell-cell adhesion (S7 Fig). Next, we mapped gene expression signatures from previously published studies of CM [25,70–76] onto our transcriptomic data and observed strong similarities with the CM transcriptomic classification proposed by TCGA [25]. Indeed, similarly to CM, we detected the ‘keratin high’ (N = 3; 27.3%) and ‘immune high’ (N = 5; 45.4%) subtypes. However, we did not identify the ‘MITF high’ subtype cluster, probably because this class is the least abundant one in CM and our rather small dataset did not have enough power to detect it (S8 Fig). The third identified cluster was characterized by overexpression of gene sets involved in cell cycle activity (S8 Fig) and by gene sets overexpressed in high-grade CM, associated with poorer survival and higher proliferation rates [72]. Patients in this class had indeed significantly higher tumor thickness (5 mm vs. 2.3 mm, p-value = 0.02, by t-test). There was no significant correlation between the transcriptomic classes and the proposed genomic classification, tumor localization or presence/absence of metastasis. In addition, such classes correlated neither with mutations in any of the known oncogenes from DriverDBv3, nor with the distribution of the deleted regions, focal or broad. Finally, no significant differences in mRNA expression were found in tarsal vs. bulbar tumors. However, in CM patients the immune subtype was associated with better survival [25] and CM patients whose expression patterns were enriched for cell cycle genes and DNA repair genes had increased metastatic risk and worse prognosis [25,77]. Therefore, projecting this information onto CJM patients could possibly provide a prognostic indication.

### Signaling pathways

Next, we integrated all SNV, CNV and RNA-seq data, to assess to which extent the ten canonical cancer signaling pathways (cell cycle, p53, Hippo, Myc, Nrf2, PI-3-Kinase/Akt, RTK-RAS, TGFβ signaling, Notch, and Wnt) [78] contribute to the pathophysiology of CJM and provide a global overview of its biology. Our analysis showed that genes in the RTK-RAS pathway were altered in all patients (14/14), by SNV or indel, and that all tested samples also showed an alteration by a CNV concordant with the gene expression level of that same gene (either an amplification/gain and significantly higher gene expression or shallow/deep deletion and significantly lower gene expression; see Methods for more details). Hippo and Wnt pathways were altered in 89% of the cases (both Hippo and Wnt: 86% by SNV/indel and 89% by a CNV). The least mutated pathway overall was NRF2, affected by CNVs in only 11% of cases (S9 Fig, S6 Table).

### Gene fusions

Lastly, we used RNA-seq data to assess the presence of possible gene fusions. Overall, 58 unique fusion events were identified. *RP11-206L10*.*9*—*PSPH*, *STEAP1*—*RAPGEF5* and *ACTG2*—*ACTG1* were detected in two individuals, as well as two known fusion events involving *PMEL*—*SQSTM1* and *PPTC7*—*HVCN1*. Interestingly, *PMEL*, encoding the promelanosome protein (P100), was involved in 4 different fusion events and fusions impacted overall 31 known cancer genes (S10 Fig). Despite *PMEL* and *MC1R* (that was deleted in 3/14 patients) are both involved in the process of pigmentation, and in turn pigmentation is associated with prognosis [79], we did not find any significant correlation between the levels of pigmentation in tumors and the presence of these molecular events.

### Conclusions

In conclusion, through the analysis of mutational load and mutational signatures, our study highlights the similarity between CJM and CM, mostly due to the effect of exposure to UV-light. Interestingly, tarsally-located CJM tumors show different behavior in terms of their genetic make-up. They carry significantly lower numbers of C>T changes, but also somatic SNVs overall, and are characterized by a different composition of mutational signatures compared to tumors localized on the light-exposed bulbar conjunctiva. This phenomenon, for which two similar tumors in the same tissue have such a different genetic background, is intriguing. Moreover, similarly to CM, CJM can be grouped into four genomic subclasses, based on their main driver events: the *BRAF*, *NF1*, *RAS* and a triple wild-type classes.

Our data also suggest the involvement of the gene *ACSS3*, not yet described in association with either CJM or CM, in CJM tumorigenesis, possibly in relationship to one of the hallmarks of cancer—the Warburg effect. In particular, the missense variant p.P532S in this gene was discovered in 3/14 of CJM patients in our cohort and in additional twelve patients and two cell lines in public databases, representing a potential hot-spot mutation. Furthermore, we showed that overexpression of the mutated (p.P532S) *ACSS3* gene leads to higher proliferative activity in the CRMM-1 cells, in line with the hypothesis of oncogenicity of this gene in CJM.

Transcriptomic classification of CJM also overlaps with that of CM. We identify three main transcriptomic subtypes: ‘cell cycle high’, ‘keratin high’, and ‘immune high’. However, we could not detect any correlation between these expression classes and the genetic profile of tumors (CNVs and/or SNVs), their metastatic status, recurrence, or patients’ outcome.

Interestingly, some of the similarities between CM and CJM described here are important indicators of the response to treatment in CM. For instance, CM patients with a high mutational load respond well to T-cell immunotherapy and patients with desmoplastic melanoma with *NF1* mutations, that are also prevalent in CJM, benefit from treatment with immune checkpoint inhibitors. While some of these treatments are being used in isolated cases, they do not yet represent the state-of-the-art therapy for advanced CJM. Similar to other rare cancers, conjunctival melanoma suffers from a lack of appropriate research, leading to many practical drawbacks for patients, such as for instance limited access to the newest treatments or ineligibility to enroll into clinical trials. By this study, we hope to provide the first basis for improving diagnostic processes and potential treatment options for all individuals affected by this disease.

## Materials and methods

### Ethics statement

This study adhered to the tenets of the Declaration of Helsinki and was approved by the “Commission cantonale d'éthique de la recherche sur l'être humain (Vaud)” and the “Ethikkommission Nordwest- und Zentralschweiz”. Prior to be enrolled in this study, all patients signed a written informed consent detailing their voluntary participation and their donation of biological material for medical research.

### DNA sequencing

The WGS data were obtained using the Complete Genomics sequencing platform [80]. Reads were aligned to the reference genome (NCBI Build 37) and somatic single nucleotide variations, indels, and CNVs were identified by comparing the matching tumor and non-tumor tissue variations, as previously described [81]. WES was performed at the iGE3 Genomics Platform in Geneva, Switzerland, using a HiSeq 4000 sequencer. Mapping was performed using NovoAlign V3.08.02 (Novocraft, Selangor, Malaysia). Mutect2 by GATK v.3.8 [67] was used for variant calling, to detect somatic single-nucleotide variants (SNVs) and indels. Oncotator v1.8.0.0 [82] was used for the annotation of all somatic variants. Coding non-synonymous variants (in the text and in Figs 1, 3, 6 -grey bars, as well as in Figs S2, S3, S4A, and S5) corresponded to those classified as: Frame_Shift_Del, Frame_Shift_Ins, In_Frame_Del, In_Frame_Ins, Missense_Mutation, Nonsense_Mutation, Nonstop_Mutation or Splice_Site in S2 Table.

Since the promoter of the *hTERT* gene was not captured by the kit used to pre-process DNA undergoing exome sequencing, we amplified it by PCR on DNA from tumor samples and analyzed it directly by Sanger sequencing, according to the procedure described by Scott *et al*. [83].

### RNA sequencing

Total RNA was extracted from tumor cells of 11 CJM patients. Size-selected transcripts were isolated for library construction and sequenced on an Illumina HiSeq 2500 at the Genomic Technologies Facility, Lausanne, Switzerland. Fastq files were mapped to the human reference genome (NCBI build 37) and the transcripts were quantified by the CLC Genomics Workbench 10.0 (https://www.qiagenbioinformatics.com/).

### CNV analysis

Somatic copy number variations (CNVs) were detected using CNVkit 0.9.5 [68] with default settings from paired tumor and normal tissues. The log2 copy number ratios were corrected using estimated purity by PureCN [79]. To call CNVs, the following thresholds were used: shallow deletion heterozygous copy number loss, deep deletion homozygous copy number loss, gain 1.5 to 2 increase and amplification > 2, increase as done previously [84]. Focal and broad CNVs were detected using the CNVkit segmentation data imported in GISTIC 2.0 [85]. The proportion of genome altered by CNV was obtained by computing the ratio between the number of base pairs of each CNV event and the total length of the genome, in each patient.

### Transcriptomic classification

The final gene expression counts from the CLC Genomics Workbench were log10 normalized and used to perform an unsupervised machine learning classification of the large-scale transcriptome profiles of the 11 patients using self-organizing maps (SOMs), by using the opoSOM 2.1.1 R package [86]. To functionally characterize obtained clusters, we overlaid GO-term ‘biological process’ and gene expression signatures from previously-published studies of cutaneous melanoma onto our data, as described by Kunz *et al*. [87].

### Fusions

STAR-Fusion [88] and EricScript [89] were used to detect fusions from RNA-seq data. According to previous studies, STAR-Fusion shows the highest sensitivity for detecting fusions [78,90]. It was therefore used as the main fusion caller, whereas the output of EricScript was integrated in the filtering process. All the fusions were annotated using FusionHub [91]. The filtering of the fusions involved: 1) exclusion of uncharacterized genes, immunoglobin genes, and mitochondrial genes, 2) exclusion of fusions from the same gene or gene paralogs, 3) exclusion of fusions present in the databases of normal samples from FusionHub, and 4) exclusion of fusions called by STAR-Fusion with a FFPM (fusion fragments per million total reads) value of less than 0.1 and simultaneously not detected by Ericscript. S10 Fig was generated using CIRCOS Table Viewer [88].

### Pathways

Genes were assigned to the ten canonical cancer pathways following the study by Vega *et al*. [78] and based on the literature. A gene was considered as impacted by an SNV or by an indel if it carried a protein-impacting variant—missense/nonsense/nonstop/splice site/frameshift insertion or deletion/change impacting start or stop codons or a change creating a *de novo* start—or any combination of these. A gene was considered as impacted by a CNV variant if there was a correlation between the CNV and the expression levels of that gene. More specifically, a gene was considered as “deleted” if its expression in individuals with deep or shallow deletions was significantly lower compared to the rest of the patients and a gene was considered as “amplified” if its expression in individuals with gains and amplifications was significantly higher compared to the rest of the patients. The CNV levels were taken from the file all_thresholded.by_genes.txt by GISTIC2.0, with the following parameters: deep deletion = -2, shallow deletion = -1, neutral = 0, gain = 1, amplification = 2. The comparisons were done using t-test with the threshold of 0.05, implemented in a custom Python (v2.7.15) script. The template for S9 Fig was generated using PathwayMapper [92].

### Plasmids

Gateway donor plasmids pDONR221 encoding the synthesized cDNA of human *ACSS3* (NM_024560), and the *ACSS3* missense mutation NM_024560: c.1594C>T/p.P532S were used as a templates in a Gateway recombination reaction (Gateway LR Clonase cat. n° 117910020) to integrate the genes in a pCDNA-DEST47 expression plasmid, under the transcriptional control of a human cytomegalovirus pCMV promoter. Recombinant plasmids were used to transform *E*. *coli* DH5α, selected on LB agar containing 100 μg/ml ampicillin. Plasmids were purified using Nucleobond extra midi-prep kit (cat. n° 740410.50, Macherey-Nagel). Purified DNA was quantified using a DropSense 96 benchtop spectrophotometer (Dropsense 96, Trinean) and integrity assessed by running the DNA on 0.8% agarose gel. Gene sequences were verified using Sanger sequencing.

### Cell culture

Conjunctival Recurrent Malignant Melanoma-1 cells (CRMM-1; RRID:CVCL_M593) were grown at 37°C, with 5% CO_2_ in a medium composed of 40% Ham's F-12K (cat. n° 21127022, Thermo Fisher), 40% RPMI 1640 (cat. n° 61870010, Thermo Fisher), supplemented with 10% Fetal Bovine Serum FBS (Lot n° BCCB2240 cat. n° F7524, Sigma-Aldrich), 6 mM Hepes and 1% Penicillin-Streptomycin solution (cat. n° P4333-100 mL, Sigma-Aldrich). Cell passages were performed every six days using Trypsin-EDTA (0.05%) (cat. n° 25300054, Thermo Fisher).

### Transfections

Transfections were performed with the Lipofectamine 2000 reagent (cat. n° 11668030, Thermo Fisher) in 6-well plates (cat. n° 92996, Techno Plastic Products AG). Cells (0.3x10^6^) were seeded to reach 80% confluence a day prior to the transfection. Lipofectamine was diluted with Opti-MEM medium (cat. n° 31985070, Thermo Fisher). After a 5-minute incubation, the lipofectamine solution was mixed with the DNA solution. The expression plasmids were diluted with Opti-MEM medium to a final volume of 150 μl, mixed with an equal volume of diluted lipofectamine solution (10 μl lipofectamine per 2.5 μg DNA) and, after 20 minutes, added dropwise to the well. Six hours after transfection, the cell medium was exchanged with fresh medium.

### Cell proliferation assay

CRMM-1 cells from three independent transfections in 6-well plates were transferred into a 96-well cell culture plate to evaluate the effect of transiently-expressed *ACSS3* alleles on cell proliferation. Six hours after transfection, adherent cells were washed with PBS and trypsinized; 20 μl cell suspension was loaded on a cell counting chamber (SD100, Nexcelom) and counted with a Cellometer Auto 1000 instrument. Ten thousand cells were seeded in 100 μl medium for each well, in a 96-well cell culture plate (cat. N° 92096, Techno Plastic Products AG). Cell proliferation assay (Cell Proliferation Kit I (MTT), cat. n° 11465007001, Roche) was performed according to the manufacturer protocol. In brief, cells were incubated with 10 μl of MTT (3-[4,5-dimethylthiazol-2-yl]-2,5-diphenyl tetrazolium bromide, 0.5 mg/ml) solution for 4 hours, the medium was removed, and purple formazan salt crystals formed during incubation were solubilized with 100 μl solubilization solution in a humidified incubator at 37°c for 2 hours. Samples were transferred into a 96 wells plate, and absorbance [A570 nm—A690 nm] was measured with an absorbance plate reader (Hidex Sense).

### RNA and Q-PCR

RNA was extracted from 0.5x10^6^ cells in 6-well plates using Illustra RNA minispin (cat n° 25-0500-70, GE Healthcare). RNA was quantified using a DropSense 96 benchtop spectrophotometer (Dropsense 96, Trinean). Two hundred nanograms RNA were used as a template for cDNA synthesis using High-Capacity cDNA reverse transcription (cat n° 4368814, Thermofisher). Gene expression was quantified on QuantStudio 3 RT-PCR system (Applied Biosystems) using the following primer sets. *ACSS3* (from plasmid): 5’-CAGGCTTCCCACCATGAAAC-3’ and 5’-TGATCTGCTCGGCAGCTTTG-3’; *ACTB* (endogenous): 5’-CACCTTCTACAATGAGCTGCG-3’ and 5’-AGCACAGCCTGGATAGCAACG-3’.

### Software information and availability

Data were analyzed with software described previously or with a combination of custom Python (v2.7.15) and R scripts (v3.6.0). To generate the figures and supplementary figures the following R packages were used:

maftools 2.0.16: Fig 1, S3 Fig, S5 Fig
ComplexHeatmap 2.0.0: Fig 3
ggplot2 3.2.1: Fig 4, S1 Fig, S4 Fig
oposSOM 2.2.0: Fig 6, S7 Fig, S8 Fig
ade4 1.7–13: Fig 2

## Supporting information

S1 Table Summary of the clinical characteristics of the 14 patients. (RxTT—Radiotherapy, PAM—Primary Acquired Melanosis, AJCC—AJCC melanoma staging system, KI67—expression of KI67, MMC—mitomycin C, OS—overall survival)
(XLSX)

S2 Table All somatic SNVs and small insertions/deletions (indels) detected in the study. (TXT)

S3 Table Summary of all the somatic variations per patient, including SNVs, Indels and CNVs. (XLSX)

S4 Table Mutations identified in the hTERT promoter. nd; not determined. (XLSX)

S5 Table All somatic CNVs detected in the study. (TXT)

S6 Table SNVs and indels impacting genes in the ten main cancer pathways, present our set of 14 CJM patients. (XLSX)

S1 Fig Expression of *APOBEC*-family genes (in Reads Per Kilobase per Million—RPKM) in CJM patients in relation to their tumor localization. Sample CM10 is shown separately, demonstrating that none of the genes is significantly overexpressed in the patient’s tumor. *APOBEC1* and *APOBEC4* were not expressed in any of the samples.
(PDF)

S2 Fig Comparison of the number of somatic variants in tarsal vs. bulbar tumors. The associated p-value for the difference in the distribution is 0.007, by t-test.
(PDF)

S3 Fig **Landscape of mutations for the genes: (A) *NF1* (B) *BRAF* (C) *HRAS* and (D) NRAS.** The grey bar represents the full protein and the colored segments visualize the positions of the specific functional domains.
(PDF)

S4 Fig Comparisons of: (A) number of somatic exonic alterations, (B) number of C>T alterations within the four genomic tumor classes defined by TCGA.
(PDF)

S5 Fig Landscape of mutations in the gene ACSS3. The grey bar represents the full protein and the colored segments visualize the positions of specific functional domains.
(PDF)

S6 Fig Recurrent focal amplifications and deletions. Recurrent focal CNVs (amplifications—right, deletions—left), as detected by Gistic 2.0, are displayed across the genome. The statistical significance of the focal events is shown as FDR q values (x axis).
(PDF)

S7 Fig Gene expression enrichment analysis of the three transcriptomic clusters. The clusters are enriched in functions related to (i) DNA replication, DNA repair and cell cycle, (ii) immune system, and (iii) keratinization, cornification and cell-cell adhesion.
(PDF)

S8 Fig Classification of CJM into an “immune high subtype”, a “keratin high subtype”, and a “cell cycle high subtype” based on the known CM gene sets. (PDF)

S9 Fig Pathways altered in CJM. (A) Proportions of altered genes in ten canonical oncogenic signaling pathways. Frequency of SNVs, amplifications and deep deletions are indicated. Color intensity reflects the frequency of the alteration. (B) Summary data per pathway, with respect to SNVs and CNVs.
(PDF)

S10 Fig Spectrum of fusion events detected in CJM. The thickness of the edges reflects the frequency of the fusions detected. Edges in green depict known cancerrelated fusion events. Genes in bold are known cancer-related genes.
(PDF)
