# Supplementary material for: Genomic and transcriptomic landscape of conjunctival melanoma
Source: PLoS Genet. 2020 Dec 31;16(12):e1009201. doi: 10.1371/journal.pgen.1009201 (PMC7775126; doi:10.1371/journal.pgen.1009201)
Supplement: S1 Fig — Sample CM10 is shown separately, demonstrating that none of the genes is significantly overexpressed in the patient’s tumor. APOBEC1 and APOBEC4 were not expressed in any of the samples. (PDF) [file pgen.1009201.s007.pdf]

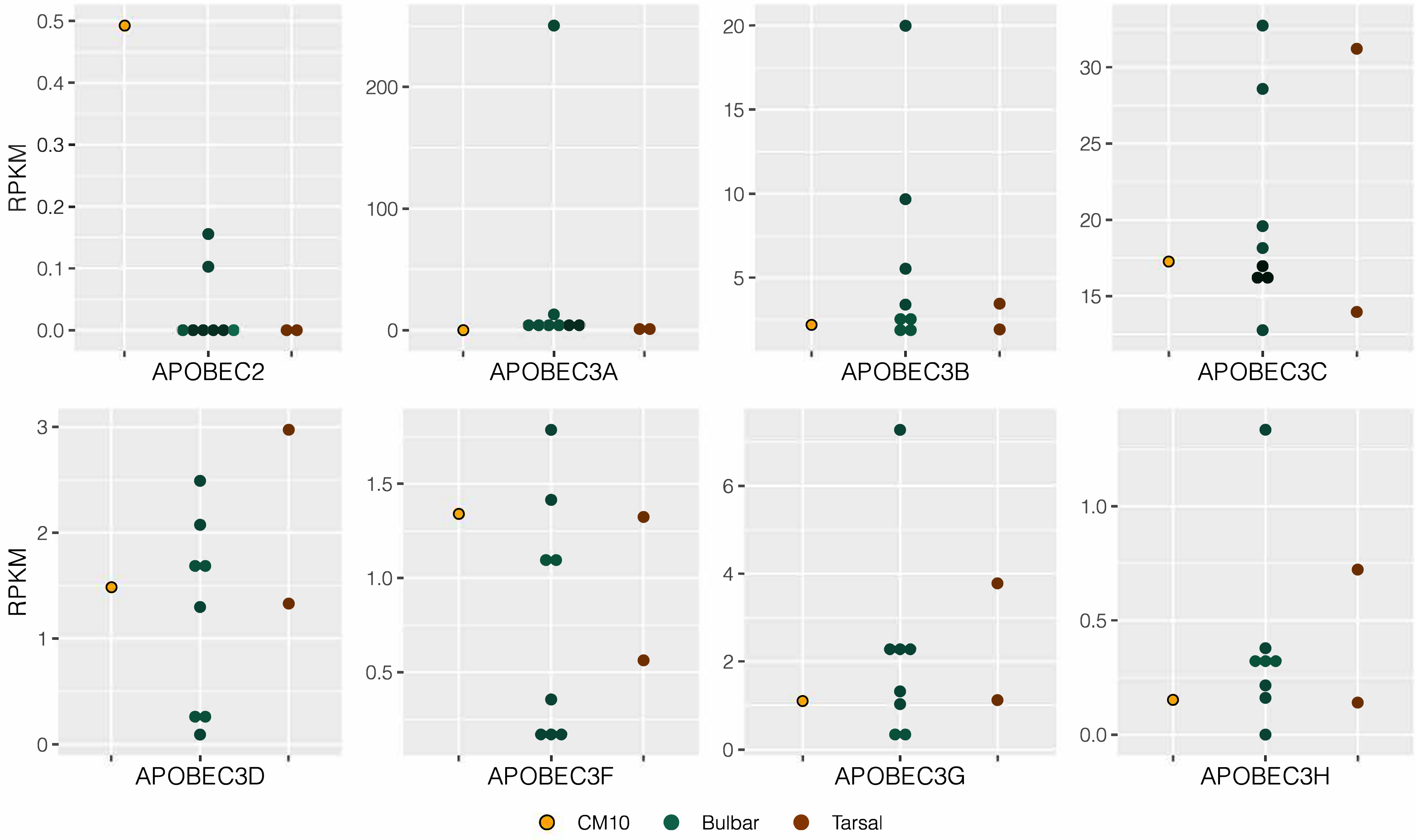

S1 Fig. Expression of APOBEC-family genes (in Reads Per Kilobase per Million - RPKM) in CJM patients in relation to their tumor localization. Sample CM10 is shown separately, demonstrating that none of the genes is significantly overexpressed in the patient's tumor. *APOBEC1* and *APOBEC4* were not expressed in any of the samples.
