# Supplementary material for: Genomic and transcriptomic landscape of conjunctival melanoma
Source: PLoS Genet. 2020 Dec 31;16(12):e1009201. doi: 10.1371/journal.pgen.1009201 (PMC7775126; doi:10.1371/journal.pgen.1009201)
Supplement: S2 Fig — The associated p-value for the difference in the distribution is 0.007, by t-test. (PDF) [file pgen.1009201.s008.pdf]

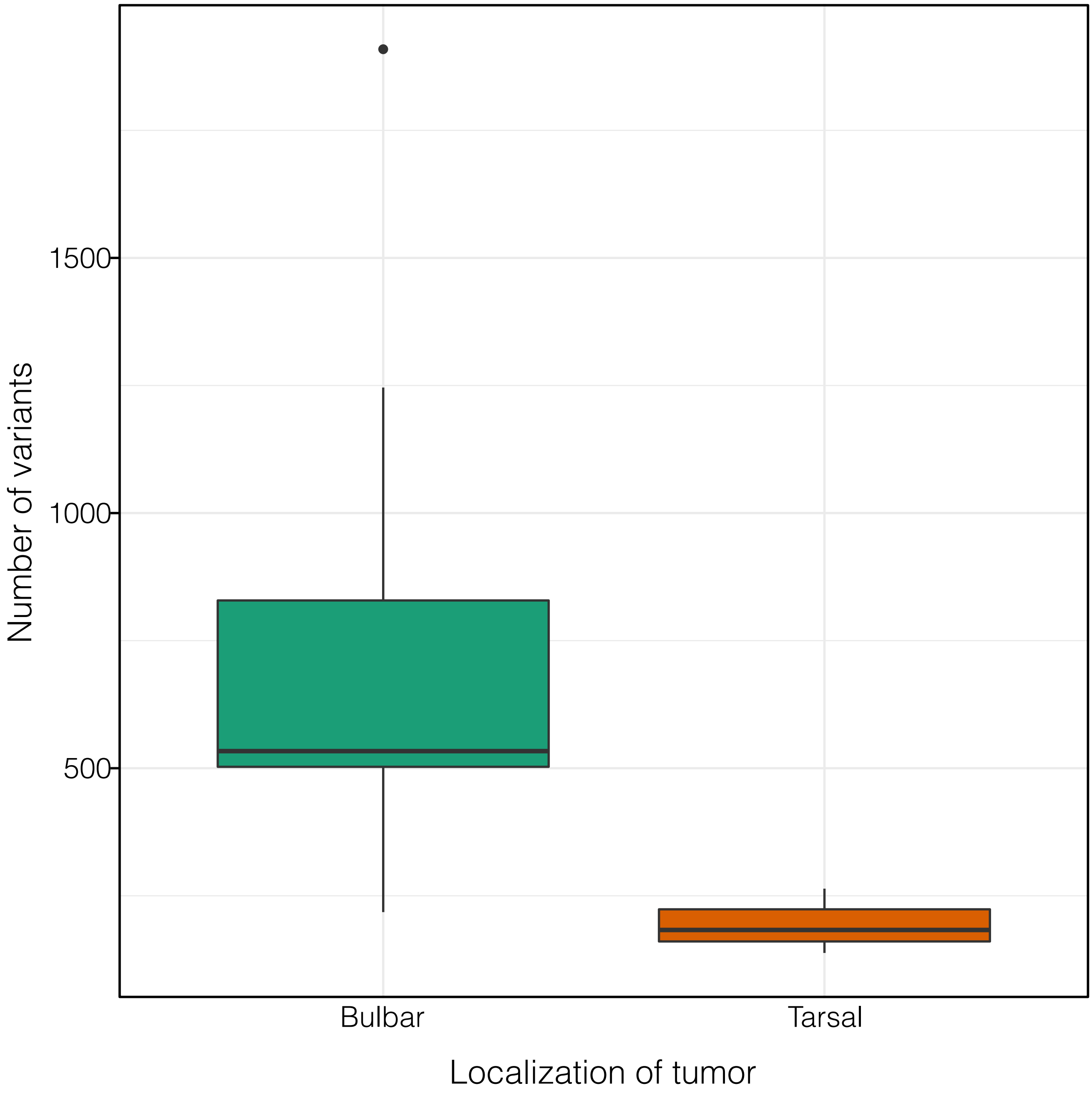

S2 Fig. Comparison of the number of somatic variants in tarsal vs. bulbar tumors. The associated p-value for the difference in the distribution is 0.007, by t-test.
