# Supplementary material for: Genomic and transcriptomic landscape of conjunctival melanoma
Source: PLoS Genet. 2020 Dec 31;16(12):e1009201. doi: 10.1371/journal.pgen.1009201 (PMC7775126; doi:10.1371/journal.pgen.1009201)
Supplement: S3 Fig — Landscape of mutations for the genes: (A) NF1 (B) BRAF (C) HRAS and (D) NRAS. The grey bar represents the full protein and the colored segments visualize the positions of the specific functional domains. (PDF) [file pgen.1009201.s009.pdf]

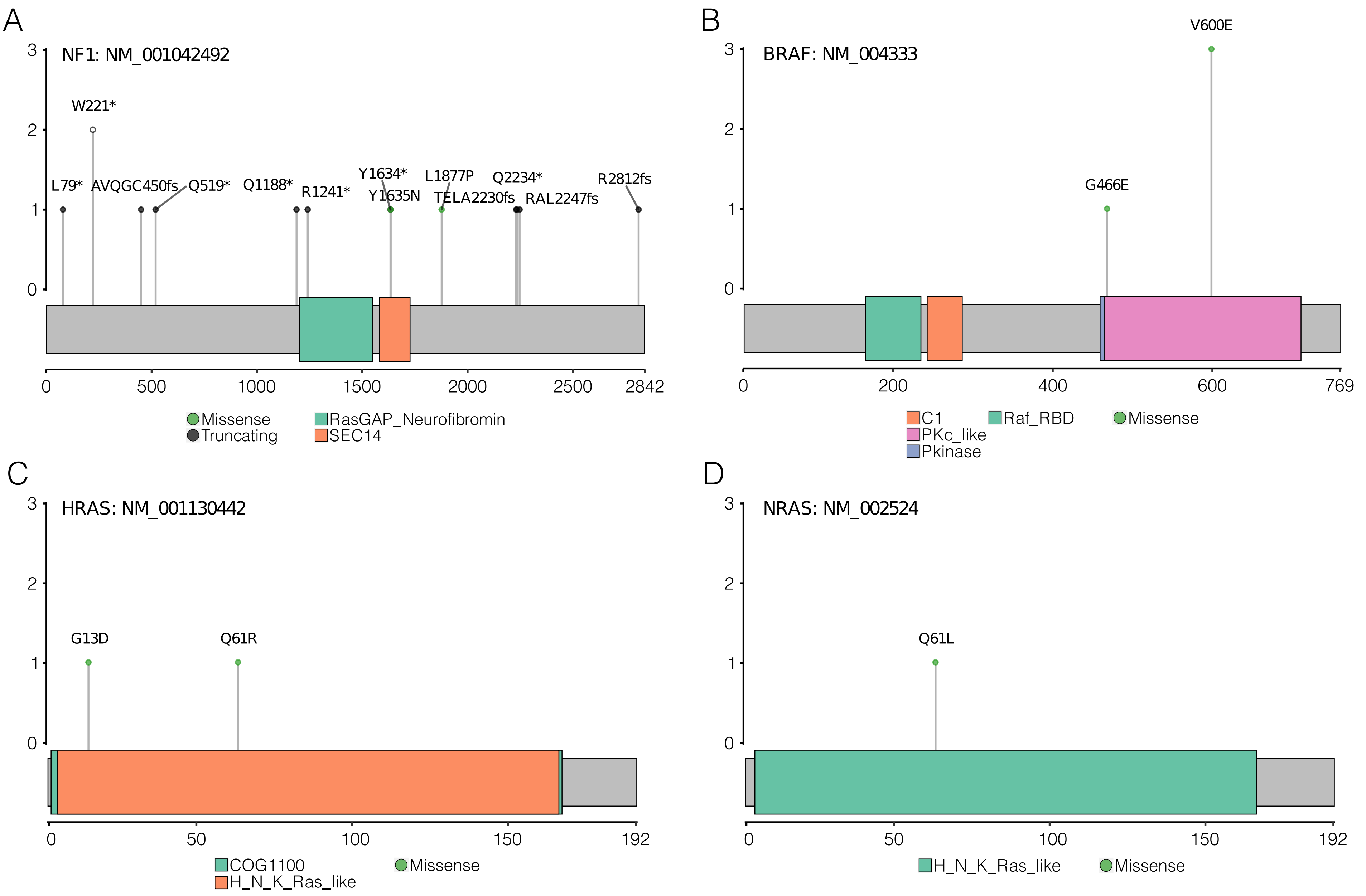

S3 Fig. Landscape of mutations for the genes: *NF1* (A), *BRAF* (B), *HRAS* (C), and *NRAS* (D). The grey bar represents the full protein and the colored segments visualize the positions of the specific functional domains.
