# Supplementary material for: Genomic and transcriptomic landscape of conjunctival melanoma
Source: PLoS Genet. 2020 Dec 31;16(12):e1009201. doi: 10.1371/journal.pgen.1009201 (PMC7775126; doi:10.1371/journal.pgen.1009201)
Supplement: S4 Fig — Comparisons of: (A) number of somatic exonic alterations, (B) number of C>T alterations within the four genomic tumor classes defined by TCGA. (PDF) [file pgen.1009201.s010.pdf]

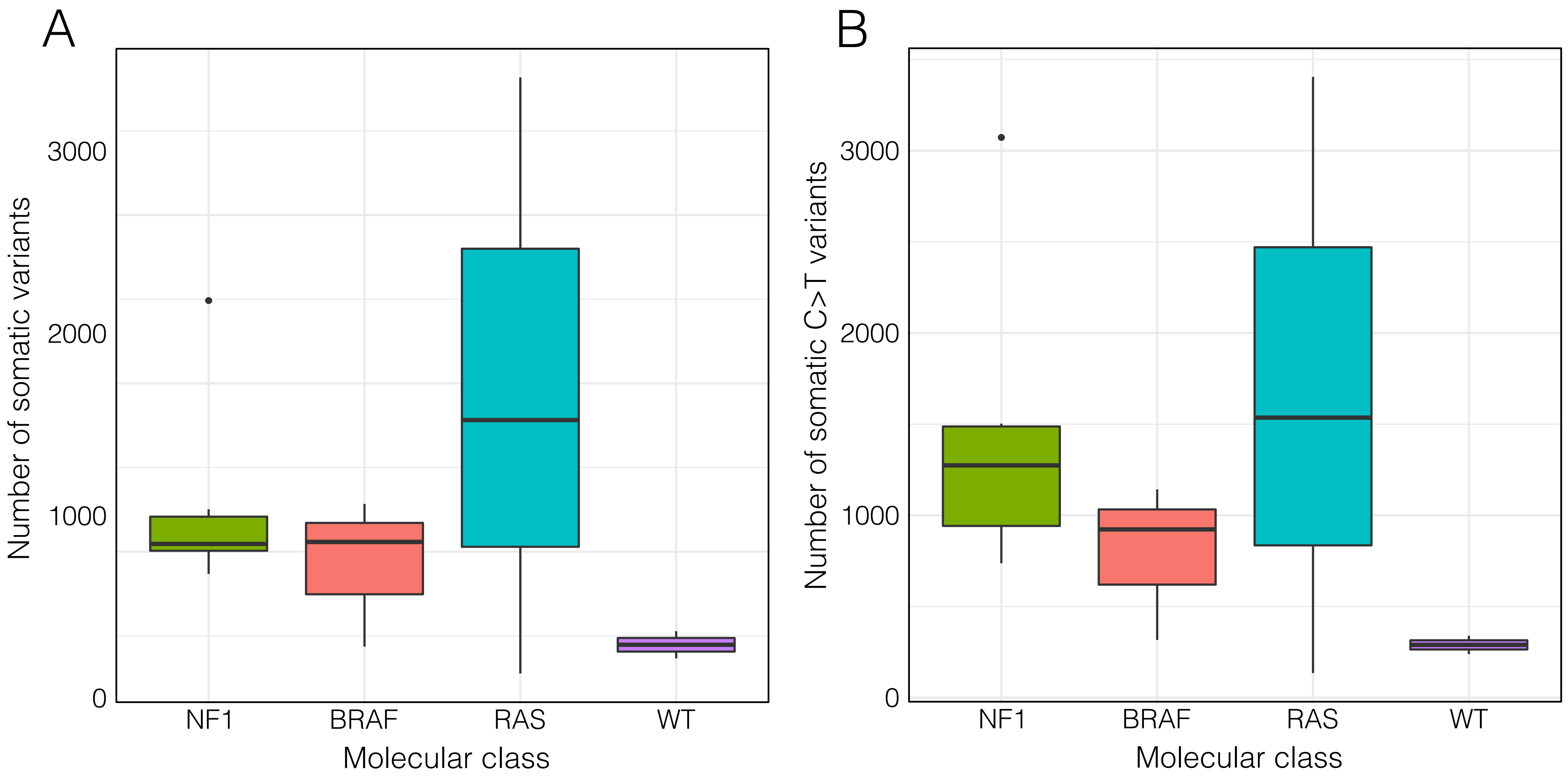

S4 Fig. Comparisons of: (A) number of somatic exonic alterations, (B) number of C>T alterations within the four genomic tumor classes defined by TCGA.
