# Supplementary material for: Genomic and transcriptomic landscape of conjunctival melanoma
Source: PLoS Genet. 2020 Dec 31;16(12):e1009201. doi: 10.1371/journal.pgen.1009201 (PMC7775126; doi:10.1371/journal.pgen.1009201)
Supplement: S5 Fig — The grey bar represents the full protein and the colored segments visualize the positions of specific functional domains. (PDF) [file pgen.1009201.s011.pdf]

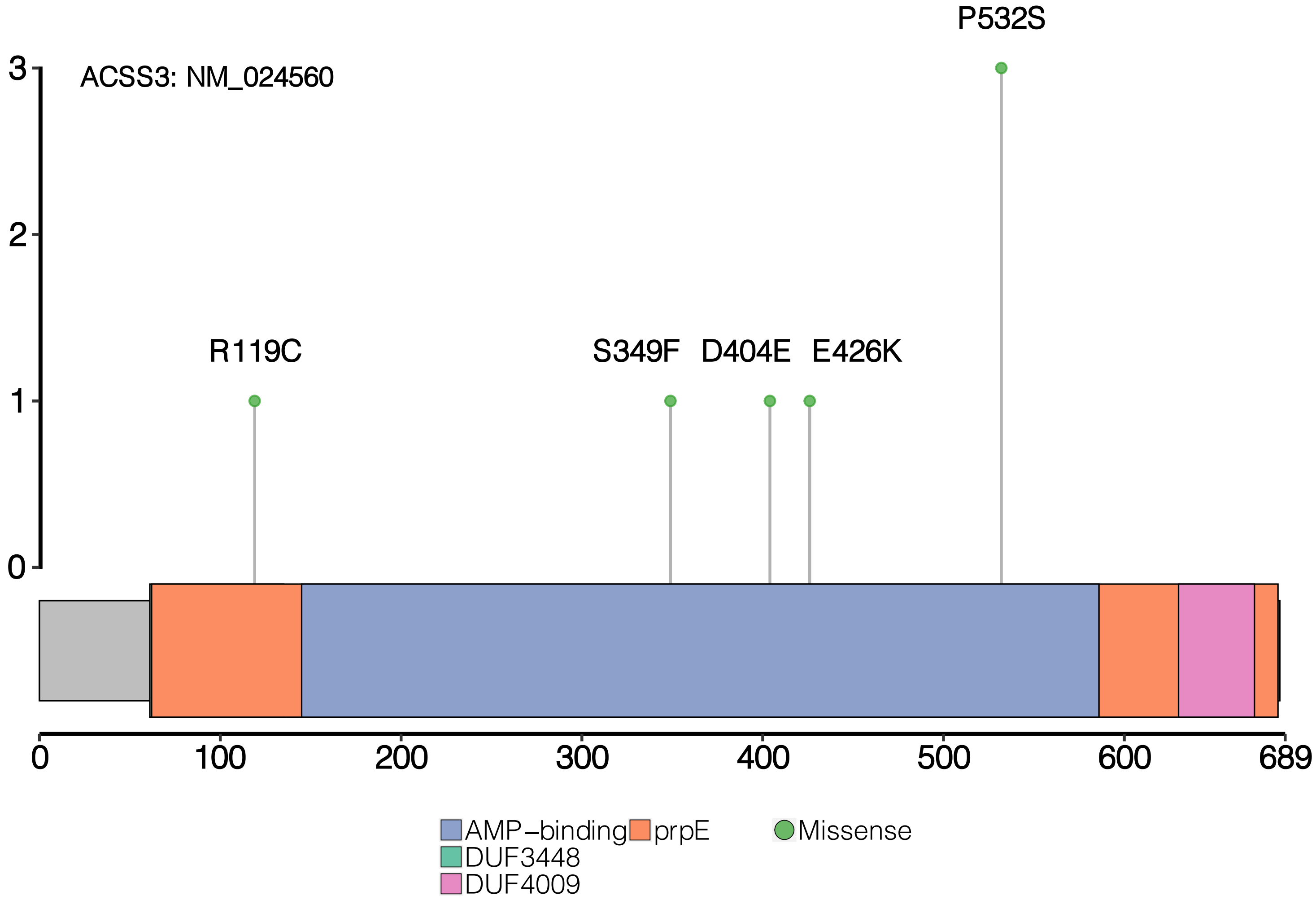

S5 Fig. Landscape of mutations in the gene *ACSS3*. The grey bar represents the full protein and the colored segments visualize the positions of specific functional domains.
