# Supplementary material for: Genomic and transcriptomic landscape of conjunctival melanoma
Source: PLoS Genet. 2020 Dec 31;16(12):e1009201. doi: 10.1371/journal.pgen.1009201 (PMC7775126; doi:10.1371/journal.pgen.1009201)
Supplement: S6 Fig — Recurrent focal CNVs (amplifications—right, deletions—left), as detected by Gistic 2.0, are displayed across the genome. The statistical significance of the focal events is shown as FDR q values (x axis). (PDF) [file pgen.1009201.s012.pdf]

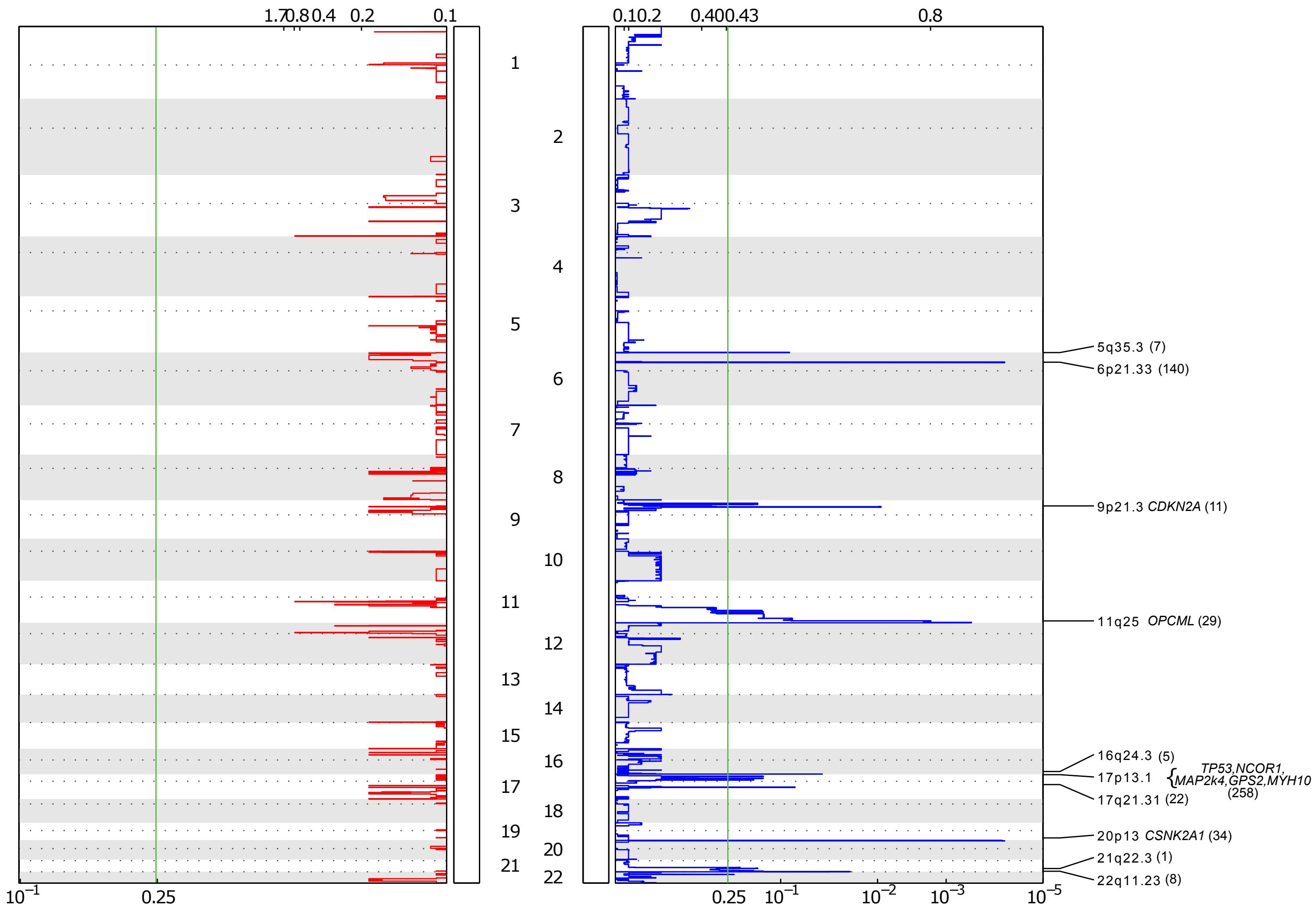

S6 Fig. Recurrent focal amplifications and deletions. Recurrent focal CNVs (amplifications - right, deletions - left), as detected by Gistic 2.0, are displayed across the genome. The statistical significance of the focal events is shown as FDR q values (x axis).
