# Supplementary material for: Genomic and transcriptomic landscape of conjunctival melanoma
Source: PLoS Genet. 2020 Dec 31;16(12):e1009201. doi: 10.1371/journal.pgen.1009201 (PMC7775126; doi:10.1371/journal.pgen.1009201)
Supplement: S7 Fig — The clusters are enriched in functions related to (i) DNA replication, DNA repair and cell cycle, (ii) immune system, and (iii) keratinization, cornification and cell-cell adhesion. (PDF) [file pgen.1009201.s013.pdf]

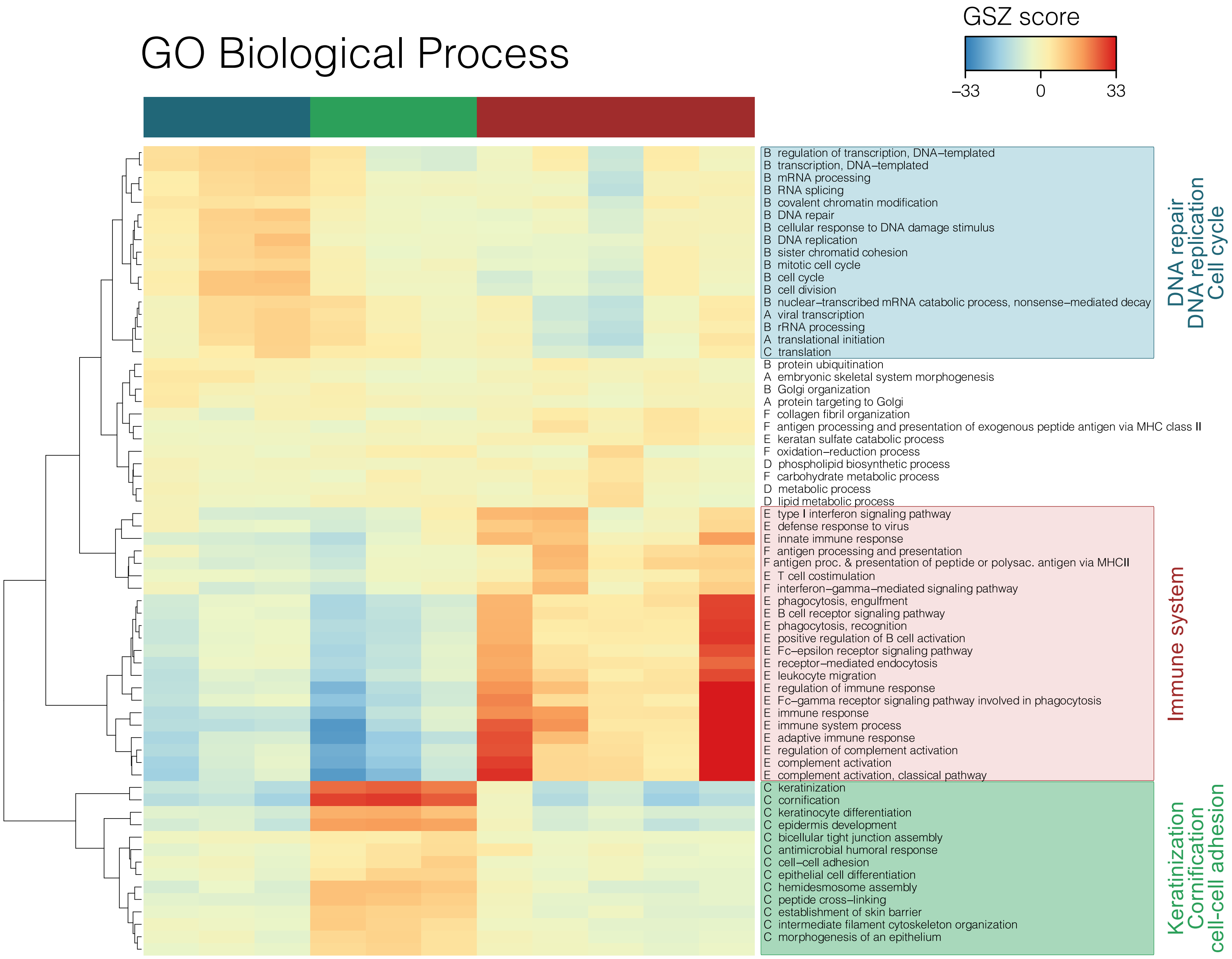


**S7 Fig. Gene expression enrichment analysis of the three transcriptomic clusters.** The clusters are enriched in functions related to (i) DNA replication, DNA repair and cell cycle, (ii) immune system, and (iii) keratinization, cornification and cell-cell adhesion.
