# Supplementary material for: Genomic and transcriptomic landscape of conjunctival melanoma
Source: PLoS Genet. 2020 Dec 31;16(12):e1009201. doi: 10.1371/journal.pgen.1009201 (PMC7775126; doi:10.1371/journal.pgen.1009201)
Supplement: S8 Fig — (PDF) [file pgen.1009201.s014.pdf]

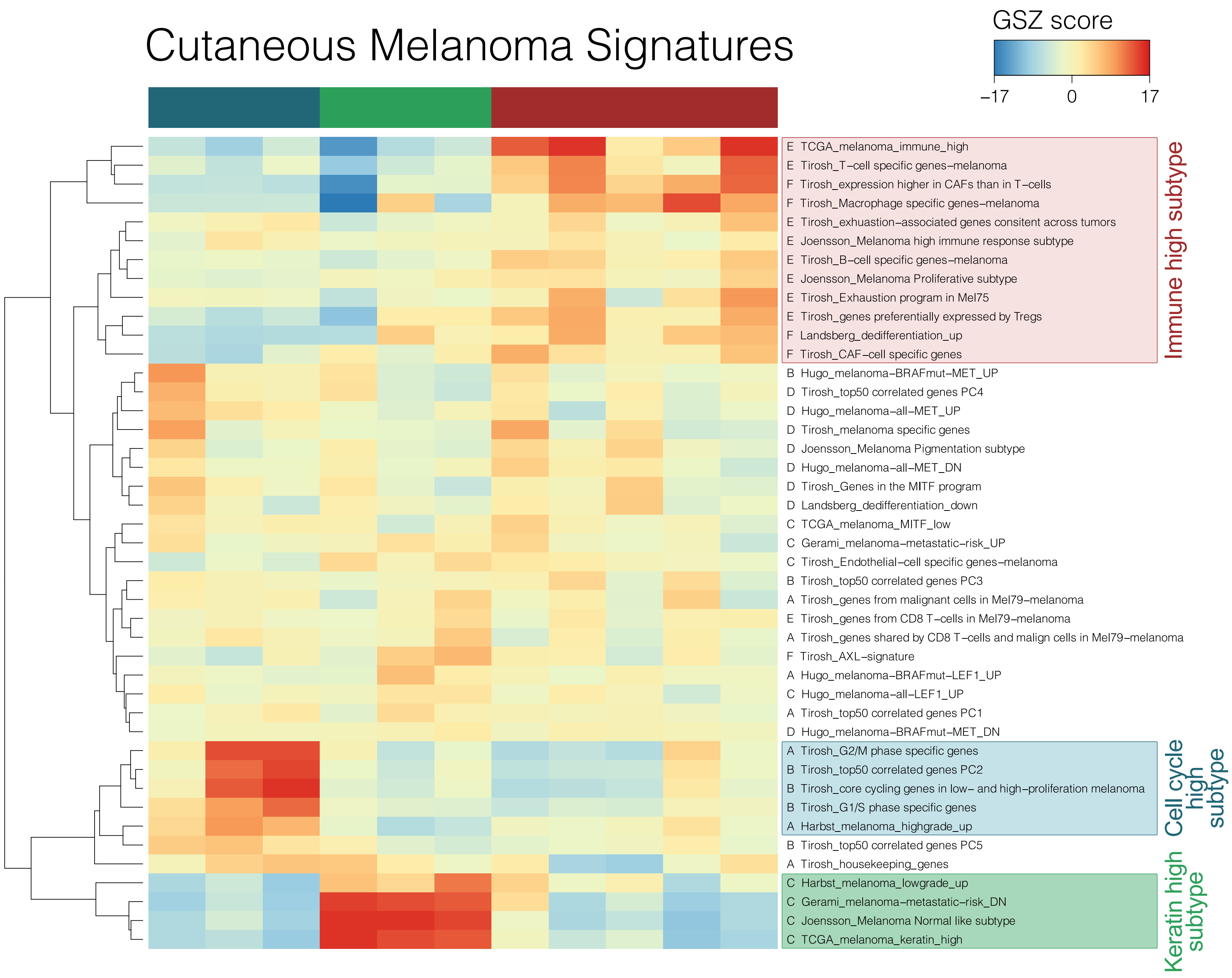

S8 Fig. Classification of CJM into an “immune high subtype”, a “keratin high subtype”, and a “cell cycle high subtype” based on the known CM gene sets.
