# Supplementary material for: Genomic and transcriptomic landscape of conjunctival melanoma
Source: PLoS Genet. 2020 Dec 31;16(12):e1009201. doi: 10.1371/journal.pgen.1009201 (PMC7775126; doi:10.1371/journal.pgen.1009201)
Supplement: S9 Fig — (A) Proportions of altered genes in ten canonical oncogenic signaling pathways. Frequency of SNVs, amplifications and deep deletions are indicated. Color intensity reflects the frequency of the alteration. (B) Summary data per pathway, with respect to SNVs and CNVs. (PDF) [file pgen.1009201.s015.pdf]

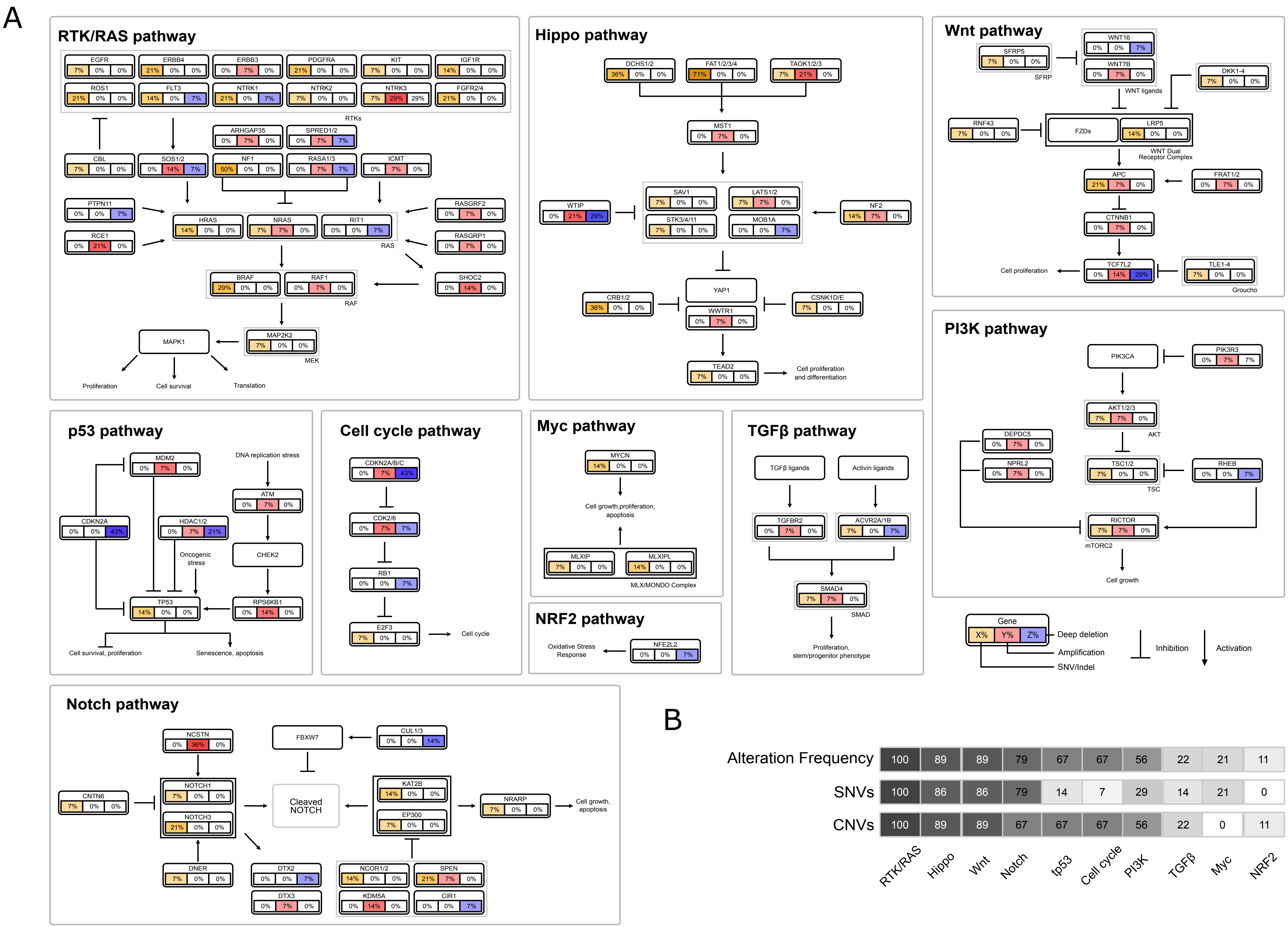

S9 Fig. Pathways altered in CJM. (A) Proportions of altered genes in ten canonical oncogenic signaling pathways. Frequency of SNVs, amplifications and deep deletions are indicated. Color intensity reflects the frequency of the alteration. (B) Summary data per pathway, with respect to SNVs and CNVs.
