# Supplementary material for: Genomic and transcriptomic landscape of conjunctival melanoma
Source: PLoS Genet. 2020 Dec 31;16(12):e1009201. doi: 10.1371/journal.pgen.1009201 (PMC7775126; doi:10.1371/journal.pgen.1009201)
Supplement: S10 Fig — The thickness of the edges reflects the frequency of the fusions detected. Edges in green depict known cancerrelated fusion events. Genes in bold are known cancer-related genes. (PDF) [file pgen.1009201.s016.pdf]

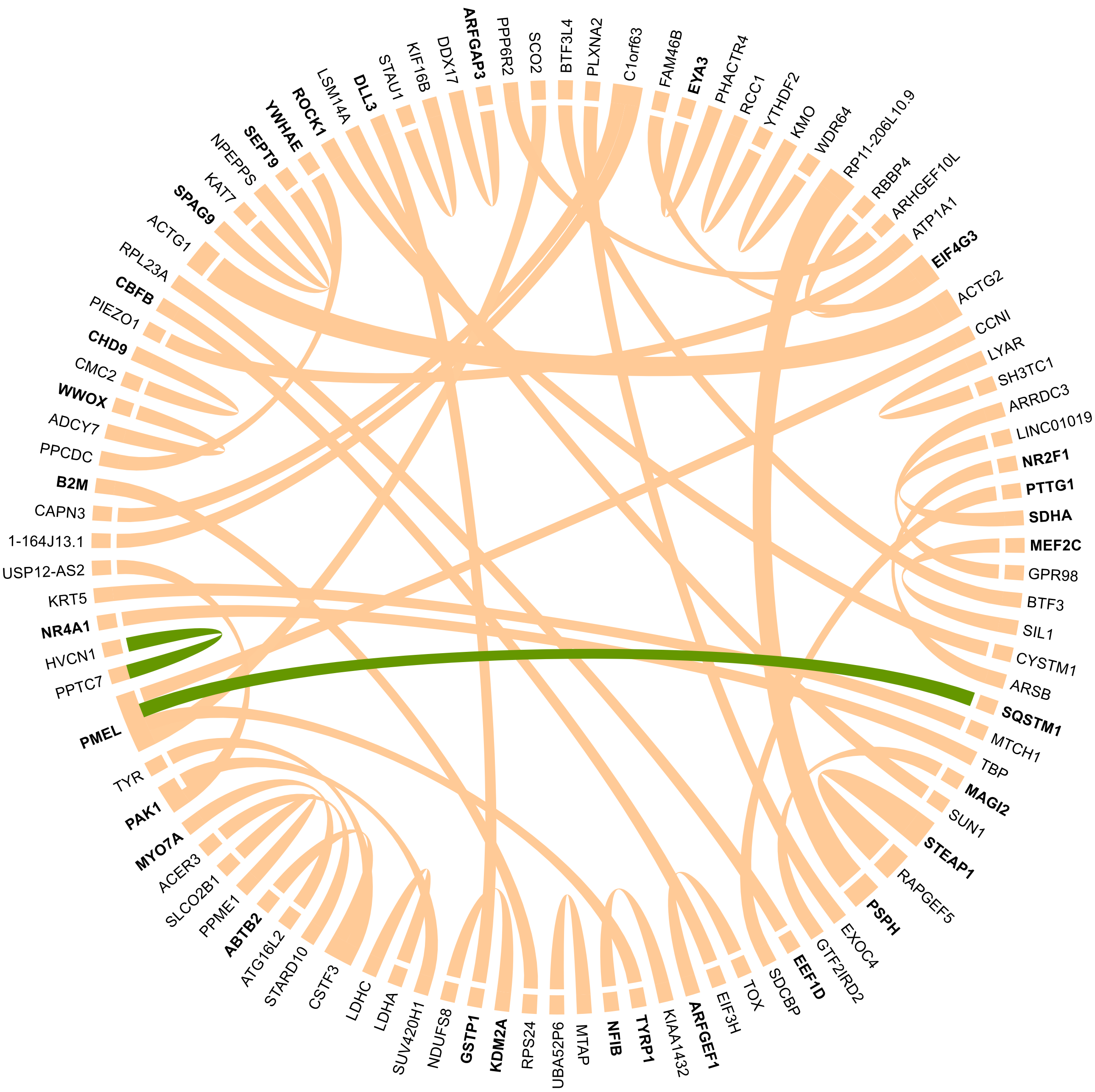

S10 Fig. Spectrum of fusion events detected in CJM. The thickness of the edges reflects the frequency of the fusions detected. Edges in green depict known cancer-related fusion events. Genes in bold are known cancer-related genes.
